# Phylogeographic patterns of a lower intertidal isopod in the Gulf of California and the Caribbean and comparison with other intertidal isopods

**DOI:** 10.1002/ece3.2599

**Published:** 2016-12-20

**Authors:** Luis A. Hurtado, Mariana Mateos, Shuang Liu

**Affiliations:** ^1^ Department of Wildlife and Fisheries SciencesTexas A&M UniversityCollege StationTXUSA

**Keywords:** Caribbean, Cirolanidae, cryptic species, *Excirolana braziliensis*, Gulf of California, Isopod, *Ligia*, *Ligia baudiniana*, *Ligia occidentalis*, marine biodiversity, sandy beach, *Tylos*, *Tylos punctatus*

## Abstract

A growing body of knowledge on the diversity and evolution of intertidal isopods across different regions worldwide has enhanced our understanding on biological diversification at the poorly studied, yet vast, sea–land interface. High genetic divergences among numerous allopatric lineages have been identified within presumed single broadly distributed species. *Excirolana mayana* is an intertidal isopod that is commonly found in sandy beaches throughout the Gulf of California. Its distribution in the Pacific extends from this basin to Colombia and in the Atlantic from Florida to Venezuela. Despite its broad distribution and ecological importance, its evolutionary history has been largely neglected. Herein, we examined phylogeographic patterns of *E. mayana* in the Gulf of California and the Caribbean, based on maximum‐likelihood and Bayesian phylogenetic analyses of DNA sequences from four mitochondrial genes (16S rDNA, 12S rDNA, cytochrome oxidase I gene, and cytochrome b gene). We compared the phylogeographic patterns of *E. mayana* with those of the coastal isopods *Ligia* and *Excirolana braziliensis* (Gulf of California and Caribbean) and *Tylos* (Gulf of California). We found highly divergent lineages in both, the Gulf of California and Caribbean, suggesting the presence of multiple species. We identified two instances of Atlantic–Pacific divergences. Some geographical structuring among the major clades found in the Caribbean is observed. Haplotypes from the Gulf of California form a monophyletic group sister to a lineage found in Venezuela. Phylogeographic patterns of *E. mayana* in the Gulf of California differ from those observed in *Ligia* and *Tylos* in this region. Nonetheless, several clades of *E. mayana* have similar distributions to clades of these two other isopod taxa. The high levels of cryptic diversity detected in *E. mayana* also pose challenges for the conservation of this isopod and its fragile environment, the sandy shores.

## 1 Introduction

The phenomenon of cryptic diversity, where a single valid species contains two or more lineages whose divergences may amount to interspecific levels, is particularly pervasive in several marine groups (Appeltans et al., 2012; Hubert et al., 2012; Knowlton, Weight, Solorzano, Mills, & Bermingham, 1993; Mathews, 2006; Moura, Harris, Cunha, & Rogers, 2008; Teske et al., 2011). Among marine animal taxa, isopods inhabiting the intertidal zone are prominent for possessing “dispersal‐limiting” traits (e.g., direct development and habitat specificity) that are expected to lead to high levels of allopatric diversification. Indeed, recent regional studies of intertidal isopods have revealed high genetic divergence among numerous allopatric lineages within what were otherwise regarded as single broadly distributed species (Hurtado, Lee, & Mateos, 2013; Hurtado, Mateos, & Santamaria, 2010; Hurtado et al., 2016; Santamaria, Mateos, Dewitt, & Hurtado, 2016; Santamaria, Mateos, & Hurtado, 2014; Santamaria, Mateos, Taiti, Dewitt, & Hurtado, 2013). This phenomenon has been reported for different intertidal isopod genera and families and in different parts of the world, implying that additional morphologically cryptic diversity is yet to be discovered in other intertidal isopod taxa and/or regions. Furthermore, the growing body of phylogeographic knowledge allows for comparison of patterns of diversification within and among basins and taxa, which will enhance our understanding on biological diversification at the poorly studied, yet vast, sea–land interface.

An abundant and common isopod taxon inhabiting intertidal and subtidal regions worldwide is the family Cirolanidae Dana, 1852 (Poore & Bruce, 2012). Recent detailed morphological studies of members of shallow‐water Cirolanidae have recognized and formally described new species (Bruce, 2004). Molecular studies of cirolanids, however, are very sparse. Nonetheless, the single named species that has been examined with some detail (i.e., *Excirolana braziliensis* Richardson, 1912) is suggested to comprise a complex of morphologically cryptic species (Hurtado et al., 2016; Sponer & Lessios, 2009; Varela & Haye, 2012). This isopod has a broad distribution along Pacific and Atlantic sandy beach coasts of the American continent. Consistent with its low dispersal potential, gene flow among geographically nearby (e.g., few kilometers) populations of *E. braziliensis* in Panama is highly restricted (Lessios & Weinberg, 1993), and populations separated by only 10s of kilometers are genetically divergent (Sponer & Lessios, 2009). The phylogeographic patterns of *E. braziliensis* reveal multiple highly divergent lineages, and multiple instances of Atlantic–Pacific divergences, some of which appear to predate the closure of the Isthmus of Panama (Hurtado et al., 2016). Whether such patterns are observed in *Excirolana mayana* (Ives, 1891), a poorly studied close relative of *E. braziliensis* that has similar distribution, biological characteristics, and morphology, is unknown.

**Figure 1:**
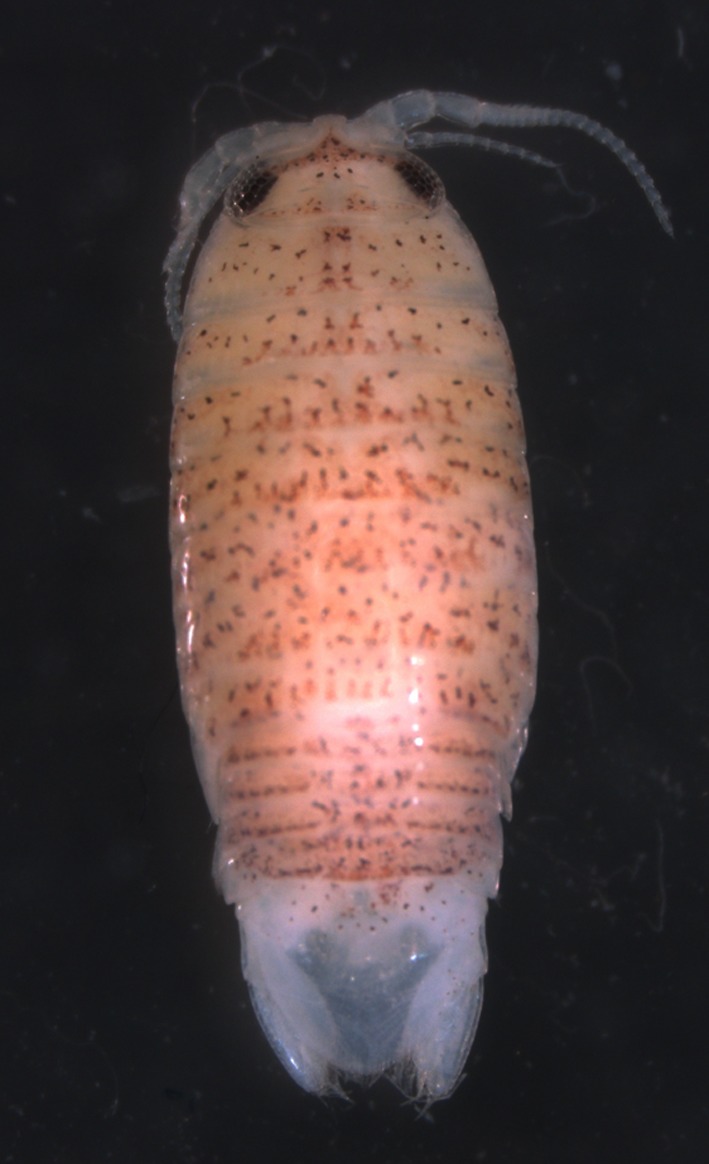
Photograph of a specimen of *Excirolana mayana* from Dzilam de Bravo (type locality), Mexico (photo by K. Conway)

*Excirolana mayana* (Figure 1) is a littoral to shallow‐water subtidal species (maximum known depth = 16 m) reported in the Pacific from the Gulf of California to Colombia and in the Atlantic from Florida to Venezuela (Brusca, Wetzer, & France, 1995). The Gulf of California, where *E. mayana* is highly abundant, is a megadiverse semi‐enclosed basin that is home to >4,900 named species of marine macro‐invertebrates (~16% are endemic; Brusca & Hendrickx, 2010; Ledesma‐Vásquez & Carreño, 2010). Nonetheless, actual diversity may be more than double, stemming from the paucity of formal species descriptions and the presence of poorly studied groups (Brusca et al., 2005). Indeed, recent genetic studies of intertidal isopods in the Gulf of California suggest that *Ligia occidentalis* (Dana, 1853) and *Tylos punctatus* (Holmes & Gay, 1909) correspond to cryptic species complexes comprised of many divergent lineages (Hurtado et al., 2010, 2013). In addition, two genetically divergent lineages of *E. braziliensis* occur allopatrically in a relatively small region of the Gulf's Baja peninsula coast (Hurtado et al., 2016). Thus, it is likely that *E. mayana* in this basin also harbors hidden diversity. Furthermore, comparison of the phylogeographic patterns of *E. mayana* in this region with those of *Ligia* and *Tylos* will shed light on the evolution of nonvagile intertidal organisms in this basin.

The Gulf of California region has a long and complex geological history that extends back to the Miocene (Helenes, Carreño, & Carrillo, 2009), aspects of which appear to be reflected in the evolutionary histories of these organisms (Hurtado et al., 2010, 2013). Compared to *Tylos* and *Ligia*,* E. mayana* has notable differences in habitat use and dispersal potential. *Tylos* and *Ligia* are mainly constrained to the supralittoral, actively avoid entering the sea, and have limited swimming and/or underwater gas exchange abilities (Brown & Odendaal, 1994; Hurtado et al., 2010; Kensley, 1974; Schultz, 1970; Taylor & Carefoot, 1990). In contrast, *E. mayana* is an agile swimmer that spends part of its life underwater in the intertidal and subtidal zones (Brusca et al., 1995), and can feed on fish carcasses (L.A. Hurtado, pers. obs.) that might be transported by currents. Therefore, *E. mayana* appears to have the highest probability of experiencing eventual long‐distance overwater dispersal. Nonetheless, phylogeographic and biogeographic patterns of *Tylos* and *Ligia* in other parts of the world indicate the occurrence of past long‐distance overwater dispersal events (Hurtado, Lee, Mateos, & Taiti, 2014; Santamaria et al., 2013, 2014). In the Gulf of California and adjacent areas, *Tylos* and *Ligia* exhibit somewhat conflicting phylogeographic patterns (Hurtado et al., 2010, 2013), which may be accounted for by differences in the extension and connectivity of the habitats occupied by *Tylos* (sandy supralittoral) and *Ligia* (rocky supralittoral) (Hurtado et al., 2013).

Characterization of phylogeographic patterns of *E. mayana* in the Caribbean will allow comparison with those reported for other intertidal isopods in this region. The Caribbean represents another region with exceptional marine diversity (Miloslavich et al., 2010) and a long and complex geological history (Iturralde‐Vinent & Macphee, 1999; Rosen, 1975). In this region, *Ligia* also harbors high levels of hidden diversity and a phylogeographic structure suggestive of a complex evolutionary history that probably has involved multiple events of long‐distance overwater dispersal (Santamaria et al., 2014). The phylogeographic patterns of *Ligia* in the Caribbean, however, neither correspond with predicted biogeographic patterns based on population connectivity of marine organisms with larval dispersal (Cowen, Paris, & Srinivasan, 2006) nor reflect a southeast to northwest colonization pattern proposed for the colonization of the Caribbean by some terrestrial animals (Hedges, 1996).

Herein, we examined phylogeographic patterns of *E. mayana* across most of its distribution using four mitochondrial markers. We conducted a thorough sampling across the Gulf of California, which enables a better understanding on the diversity and evolutionary history of this isopod in this megadiverse basin. We also examined specimens of *E. mayana* from the Caribbean, which albeit scattered, represent different regions across this basin, thereby providing insights into the diversity and evolution of *E. mayana* in this basin as well. Given its biology, and what has been observed in other intertidal isopods, we expected to uncover high levels of genetic diversity in both regions. We compared the phylogeographic patterns of *E. mayana* with those of *Ligia* and *E. braziliensis* (Gulf of California and Caribbean) and *Tylos* (Gulf of California). The results add to the growing body of knowledge on the diversity and evolution of intertidal isopods across different regions worldwide.

## 2 Methods

### 2.1 Samples

**Figure 2:**
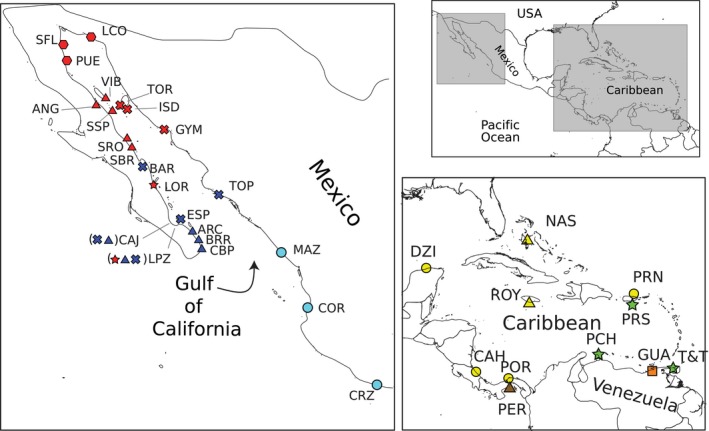
Sampled localities for *Excirolana mayana*. Shaded rectangles in small panel indicate the enlarged Gulf of California and Caribbean regions of the other panels. Color/shape‐coding and/or locality abbreviation correspond with other figures and tables. Detailed information for each locality is presented in Table S1. Map source: Administrative units (admin.shp). Edition 10.1. ArcWorld Supplement, 2012. Basemap created by Cecilia Smith using ArcGIS. Version 10.3 Redlands, CA: Esri, 2014

We collected individuals with diagnostic morphology of *E*. *mayana* from 23 localities across the Gulf of California (Figure 2). Although the locality Carrizalillo (CRZ) is not within the Gulf of California in the strict sense, we hereafter treat it as such for practical purposes. We also included individuals with diagnostic morphology of *E*. *mayana* from Dzilam de Bravo (type locality), Venezuela, Aruba, Puerto Rico, Trinidad, Jamaica, Bahamas, Panama, and Costa Rica (Figure 2). As out‐groups, we added samples of *E. braziliensis* from across the Americas, *Excirolana hirsuticauda* (Menzies, 1962) from Chile and *Excirolana chiltoni* (Richardson, 1905) from Korea and Oregon (Hurtado et al., 2016).

### 2.2 Molecular methods

Genomic DNA was extracted from leg tissue with the DNEasy kit (Qiagen, Inc., Valencia, CA, USA). Four mitochondrial gene fragments were amplified: 16S ribosomal (r) DNA; 12S rDNA; cytochrome b gene (Cytb); and cytochrome oxidase I gene (COI). The 16S rDNA and 12S rDNA primers (Podsiadlowski & Bartolomaeus, 2005) amplify fragments of 439 and 480 bp, respectively. The primers for the COI gene fragment (710‐bp) were modified from the COI primers in the study of Folmer, Black, Hoeh, Lutz, and Vrijenhoek (1994) by addition of an M13 tail (Table S4). The Cytb primers (Merritt et al., 1998) amplify a fragment of approximately 430 bp. We used exonuclease and shrimp alkaline phosphatase to clean PCR‐amplified products, which were subsequently sequenced at the University of Arizona Genetics Core. We used Sequencher 4.8 (Gene Codes, Ann Arbor, MI, USA) to edit sequences and remove primers. To examine genetic variation within localities, we obtained Cytb sequences from three to five individuals per locality for most localities. As most localities exhibited little variation among individuals (see section 3), only one individual was further characterized for the additional genes. For the few localities in which divergent Cytb haplotypes were obtained, we further characterized one representative per divergent haplotype.

### 2.3 Sequence alignment and phylogenetic analyses

Sequence alignments were performed with the online version of MAFFT v.6.0 (Katoh & Toh, 2008). Because there was little difference between L‐INS‐I strategy and Q‐INS‐I strategy, we chose the former one. MacClade v.4.06 (Maddison & Maddison, 2005) was used to check for premature stop codons in protein‐coding genes. We used the online version of GBlocks v.0.91b (Castresana, 2000) to identify positions that should be excluded due to questionable homology. The following parameters were assumed: “Allowed Gap Positions” = half; “Minimum Length of A Block” = 0.5 or 10; and “Maximum Number of Contiguous Nonconserved Positions” = 4 or 8 for removing uncertain homology sequence blocks.

Datasets of each separate gene were analyzed so as to assess genetic diversity within localities (i.e., Cytb; Dataset S2) and select individuals to be sequenced for additional genes, measure genetic distances by gene, and conduct phylogenetic analyses without missing data—the 12S rDNA dataset (Dataset S1) allowed the inclusion of the specimen from the type locality (DZI) and the *E. mayana* sequence from Pacific Panama (PER) reported by Sponer and Lessios (2009); the 16S rDNA dataset (Dataset S3) also allowed inclusion of the DZI specimen (i.e., we were unable to obtain the COI and Cytb sequences for this specimen). The four mitochondrial genes were concatenated into a dataset that included one *E. mayana* representative per locality (or more when the Cytb results suggested more than one divergent haplotype occurred at a locality; i.e., LPZ and CAJ), as well as all out‐groups (i.e., *E. braziliensis*,* E. hirsuticauda*, and *E. chiltoni*). We used the program jModeltest v0.1.1 (Posada, 2008) to select the most appropriate DNA substitution model among 88 candidate models computed from a fixed BioNJ‐JC tree. We used the following three criteria: the Akaike information criterion (AIC), corrected AIC(c), and Bayesian information criterion (BIC; Table S2).

We conducted maximum‐likelihood (ML) and Bayesian analyses. ML analyses were performed in RaxML v. 8.0.7, which implements the Rapid Bootstrap followed by ML search (Stamatakis, 2014). We also used GARLI v.2.0 (Zwickl, 2006) with bootstrap searches, which uses genetic algorithms for the ML search. Bootstrap trees were summarized (50% majority‐rule consensus) with the SumTrees script of DendroPy v.3.10.1 (Sukumaran & Holder, 2010). Bayesian analyses were performed within MrBayes v.3.2.1 (Huelsenbeck & Ronquist, 2001; Ronquist & Huelsenbeck, 2003; Ronquist et al., 2012) and Phycas v.1.2.0 (Lewis, Holder, & Swofford, 2010), implementing the polytomy prior (Lewis, Holder, & Holsinger, 2005) in the latter. The polytomy prior is one of the proposed ways to address problems arising from the “star‐tree paradox” (Lewis et al., 2005). For the MrBayes analyses, we used 10 billion generations, and collected a tree every 1,000 generations. In both, ML and Bayesian analyses, we used the closest more complex model available if the best models identified by jModeltest could not be implemented (Tables S2 and S3). Otherwise, when the combination of a proportion of invariable sites (I) and a Gamma distribution of rates among sites (G) was selected as the best model, the parameter (I) was removed to avoid the potential problems related to dependency between two parameters (see RaxML manual and Yang, 2006). In addition to the single‐partition analyses described above, we conducted a multiple partition MrBayes analysis, implementing the best partitioning scheme identified by PartitionFinder v.1.0 (Lanfear, Calcott, Ho, & Guindon, 2012). For noncoding genes, each gene was set as one partition, whereas for coding genes, each gene and codon was set as a separate partition. The following parameters were used in PartitionFinder: branch lengths = linked; models = all; model selection = BIC; search = greedy. The selected partitioning schemes are shown in Table S3.

Convergence and adequate sampling of the posterior distribution were assessed by the following: (1) stable posterior probability values converged in more than 200 samples in Tracer v.1.5 (Rambaut & Drummond, 2007); (2) a high correlation between the split frequencies of independent runs as implemented in AWTY (Nylander, Wilgenbusch, Warren, & Swofford, 2008); (3) small and stable average standard deviation of the split frequencies of independent runs; (4) potential scale reduction factor close to 1. Samples prior to reaching a stationary posterior distribution were eliminated as burn‐in (Table S3).

Pairwise genetic distances were estimated for the COI gene and 12S rDNA gene separately with the Kimura‐2‐parameter (K2P) correction implemented in MEGA v.5 (Tamura et al., 2011). For the 12S rDNA gene, pairwise genetic distances were estimated on the dataset that excluded positions of questionable homology, and using pairwise deletion for missing data.

## 3 Results

**Figure 3:**
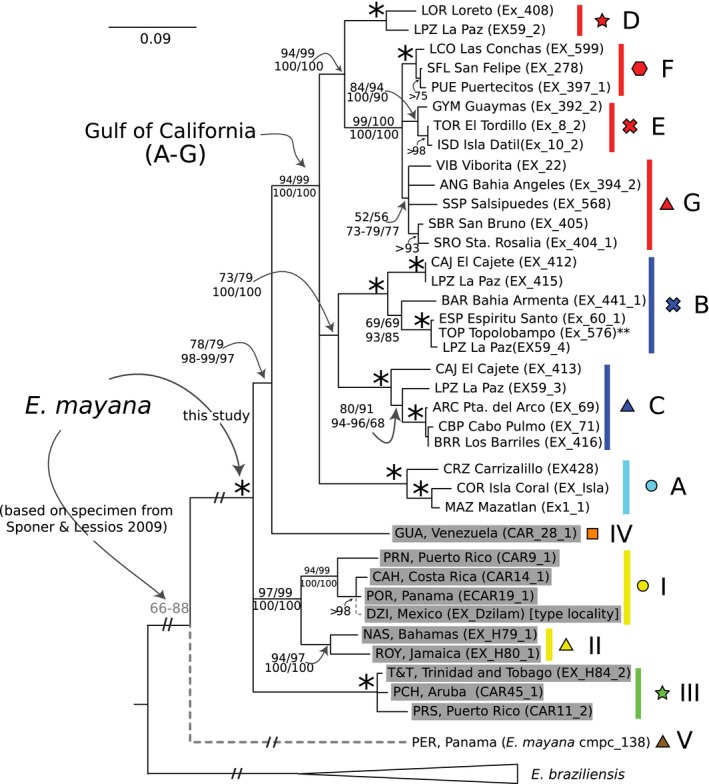
RaxML bootstrap majority‐rule consensus tree inferred with four of the concatenated mitochondrial genes (12S + 16S + Cytb + COI). Tree was rooted with *Excirolana chiltoni* and *Excirolana hirsuticauda* (not shown). *Excirolana braziliensis* lineage is shown collapsed. Taxon names with gray background represent specimens from the Caribbean. Colors, shapes, and three‐letter taxon id abbreviation match those in Figure 2. Colors denote clades/lineages, whereas different shapes denote subclades. Clade support values (>50% bootstrap or posterior probability) are shown (from left/top to right/bottom respectively: Garli, RaxML, MrBayes (one partition and “best partition” scheme), and Phycas (see Table S3). *Denotes clades that received 100% support with all analyses. Clades with a single support number had at least the value shown in all analyses. **Denotes sample that was redrawn on tree based on Cytb (see Fig. S1) and 12S rDNA results. Dashed branch indicates position of specimen morphologically assigned to *Excirolana mayana* from the Pacific coast of Panama on the basis of a portion of the 12S rDNA gene (GenBank Acc. No. KP184702; Sponer & Lessios, 2009; support based on RaxML bootstrap values). Phylogenetic position of specimen from type locality (DZI, Dzilam de Bravo) was inferred based on the 12S and 16S rDNA gene regions

All new sequences from this study have been deposited in GenBank under accession numbers KX530937, KX530941, and KT122410–KT122766. The dataset comprised of the concatenated fragments from four mitochondrial genes (12S rDNA, 16S rDNA, Cytb, and COI) and all out‐groups included 57 taxa and 1,512 characters, of which 604 were parsimony informative (Table S2; Dataset S4). Based on this dataset, individuals with the diagnostic morphology of *E. mayana* formed a well‐supported clade (100 BS; 100 PP; Clades I–IV + A–G) to the exclusion of *E. braziliensis* (Figure 3; tree was rooted with *E. hirsuticauda* and *E. chiltoni*; not shown). In addition, based on the 12S rDNA dataset alone, a previously published sequence from the Pacific coast of Panama morphologically assigned to *E. mayana* appeared as a divergent (15.5%–19.1% K2P; Table 1) sister to the remaining *E. mayana* sequences in this study (overlaid with a dashed branch; lineage V; Figure 3), with clade support of 66%–88%. Within the clade comprised of the remaining *E. mayana* lineages, a basal trichotomy was recovered: Clade I + II; Clade III; and lineage IV + a clade with the samples from the Gulf of California (A–G). Clade I + II (97–99 BS; 100 PP) was comprised of the western Caribbean islands of Jamaica and Bahamas (Clade II), and its sister (Clade I), comprised of northern Puerto Rico, northern Yucatan Peninsula (i.e., the type locality Dzilam de Bravo), and the Caribbean side of Panama and Costa Rica. COI K2P divergences between Clade I and Clade II were 11.4%–15.9% (Table 1). Clade III was comprised of samples from three eastern Caribbean islands: Trinidad; Aruba; and southeastern Puerto Rico. The COI K2P genetic divergence between Clade III (western Caribbean) and Clade I + II was 19.4%–22.2% (Table 1). The monophyly of the Gulf of California clade was highly supported (94–99 BS and 100 PP; Figure 3), and the sample from Venezuela (IV in Figure 3) appeared as its closest relative (78–79 BS and 97–99 PP). The COI K2P genetic divergence between the Gulf of California and the lineage from Venezuela was 17.5%–20.6% and between Venezuela + Gulf of California versus Clades I–III was 14.6%–28.5%.

**Table 1:** Ranges of percent Kimura‐2‐parameter distances among the main *Excirolana mayana* clades in the Caribbean region analyses, the Gulf of California clade (divergence within this clade are shown in Table 2), and the out‐group taxa

	I	II	III	IV	V	*E. mayana* Gulf of California	*Excirolana chiltoni* Korea	*E. chiltoni* USA	*Excirolana hirsuticauda*
I	1.2/9.5	11.4–15.9	19.4–21.4	16.1–17.1	NA	16.7–28.5	23.0–25.8	20.6–22.4	26.5–27.0
II	3.1–6.3	3.7/7.1	20.5–22.2	17.3–18.1	NA	14.6–21.3	22.7–23.5	21.4–22.6	28.2–28.7
III	7.7–11.3	8.5–15.7	3.8/4.4	20.9–22.8	NA	19.2–27.3	27.3–28.5	21.1–24.0	27.5–28.3
IV	9.1–9.9	7.5–10.6	8.3–10	NA	NA	17.5–20.6	25.5	22.2	29.1
V (Panama Pacific)	17.6–17.7	17.8–18.0	16.8–19.1	16.6	NA	NA	NA	NA	NA
*E. mayana* Gulf of California	6.3–10.6	5.7–11.9	9.0–12.8	6.6–9.1	15.5–17.8	6.4/21.5	23.9–29.3	19.3–24.1	22.9–30.7
*E. chiltoni* Korea	15.6	16.7–17.5	19.3–20.3	17.5	14.3	11.3–13.9	NA	18.4	25.3
*E. chiltoni* USA	21.2–21.3	21.4–23.3	19.2–22.2	15.6	15.4	18.3–20.3	8.3	NA	21.2
*E. hirsuticauda*	17.1–17.6	17.4–17.7	24.9–28.4	20.7	18.1	23.5–26.1	14.7	16.5	NA

Lower matrix: 12S rDNA gene fragment distance. Upper matrix: COI gene distance. Values on diagonal show maximum within‐clade divergence (left: 12S rDNA gene fragment; right: COI gene).

The Gulf of California clade was split into three clades effectively joined at a basal trichotomy. The first one, Clade A (100 BS and PP), was restricted to mainland Mexico from the southern Gulf of California (Mazatlan) to southern Mexico (Playa CRZ; Figure 2). Clade A was 13.6%–21.0% divergent (COI K2P; Table 2) from the remaining Gulf of California lineages. The maximum COI K2P divergence within Clade A was 13.1% (Table 2). The second basal lineage (Clade BC; 73–79 BP; 100 PP) was comprised of the sister clades B and C. Clade B (100 BS and PP) was found mainly in Bahia La Paz (i.e., La Paz, El Cajete, and Espiritu Santo), in the Baja Cape region, in one peninsular locality further north in the central peninsula (i.e., Armenta, Bahia Concepcion), and in the Gulf mainland locality of Topolobampo. Clade C (100 BS and PP) was restricted to the Cape region, and its distribution overlaps with that of Clade B in the area of Bahia La Paz. The COI K2P divergence of Clade B versus C was 13.4–19.6% (Table 2). Clade B was divided into three lineages (CAJ‐LPZ, BAR, and ESP‐TOP‐LPZ; maximum COI K2P divergence among them was 9.7%), two of which had representatives in La Paz. The samples from Topolobampo (mainland locality) were identical to samples from Espiritu Santo based on Cytb (Fig. S1) and 12S rDNA sequences (not shown). Clade C was also divided into three lineages (CAJ, LPZ, and ARC‐CBP‐BRR; maximum COI K2P divergence among them was 10.5%); two occurred in Bahia de La Paz and one south of this bay (i.e., Puerta del Arco, Los Barriles, and Cabo Pulmo).

**Table 2:** Ranges of percent Kimura‐2‐parameter distances among the main *Excirolana mayana* clades in the Gulf of California region (Figure 3)

	A	B	C	D	E	F	G
A	2.8/13.1	17.4–20.8	13.6–19.4	14.1–18.0	16.5–18.8	16.2–19.8	16.5–21.0
B	3.8–5.0	2.5/9.7	13.4–19.6	16.4–21.1	17.4–20.6	18.1–19.9	18.4–21.5
C	5.7–6.4	3.1–5.0	1.2/10.5	16.7–19.0	16.0–19.3	15.8–20.5	16.9–20.8
D	4.4–5.5	1.8–3.7	1.8–3.8	1.2/7.3	14.5–16.0	14.7–15.4	14.0–16.9
E	5.0–5.1	1.8–3.7	1.2–1.8	0.6–1.8	0/3.2	5.2–6.2	5.1–7.1
F	5.0–5.1	1.8–3.7	1.2–1.8	0.6–1.8	0	0/2.8	5.0–6.8
G	5.0–5.7	1.2–2.5	3.0–3.9	0.6–1.8	0–0.6	0–0.6	0.6/3.8

Lower matrix: 12S rDNA gene fragment distance. Upper matrix: COI gene distance. Values on diagonal show maximum within‐clade divergence (left: 12S rDNA gene fragment; right: COI gene).

The third basal lineage within the Gulf of California clade is Clade DEFG (red in Figures 2 and 3; 94–99 BS and 100 PP). Within this clade, Clade D (100 BS and PP) was found in two southern Baja localities (Loreto and La Paz), whereas its sister, Clade EFG (≤99 BS and PP), was the only *E. mayana* lineage found in the northern half of the Gulf, along both the mainland and Baja peninsula coasts. Divergence between clades D and EFG was 14.0%–16.9% (COI K2P; Table 2). Within Clade EFG, a basal trichotomy was recovered. Clade E (84–94 BS and 90–100 PP) was found in the eastern central part of the Gulf (i.e., Guaymas, Isla Datil, and Isla Tiburon). Maximum COI K2P divergence within Clade E was 3.2%. Clade F (100 BS and PP) was found at the northernmost part of the Gulf (i.e., Puerto Peñasco, San Felipe, and Puertecitos). Maximum COI K2P divergence within Clade F was 2.8%. Clade G received relatively low support (52–56 BS; 73–79 PP) and was found in the mid‐Gulf on the western Mid‐riff islands and the Baja peninsula. Within this clade, the samples from Santa Rosalia and San Bruno formed a monophyletic group (>93 BS and PP). Maximum divergence within Clade G was 3.8% (COI K2P).

### 3.1 Mitochondrial diversity within localities

Except for the localities in which more than one divergent lineage occurred (e.g., La Paz, El Cajete), relatively low Cytb diversity was found within localities (Fig. S1). In addition, sharing of identical or highly similar Cytb haplotypes within clade and between localities was observed in several instances, for example, Mazatlan, Playa CRZ, and Isla Coral (Clade A); La Paz and El Cajete (Clade B); Topolobampo, La Paz, and Espiritu Santo (Clade B); Santa Rosalia and San Bruno (Clade E–H).

## 4 Discussion

Despite its broad distribution and abundance, *E. mayana* has received little attention. Our study is the first to examine genetic diversity and the evolutionary history of this isopod. As expected from its biological characteristics, high levels of hidden genetic differentiation were detected within *E. mayana* sampled from the Gulf of California and the Caribbean (i.e., the highest K2P divergence at COI was ~28.5%). Similar patterns of previously unknown diversity have also been reported in its close relative *E. braziliensis* (Hurtado et al., 2016), and the coastal isopods *Ligia* and *Tylos*, in these and other regions (Eberl, Mateos, Grosberg, Santamaria, & Hurtado, 2013; Hurtado et al., 2010, 2013, 2014; Santamaria et al., 2013, 2014).

### 4.1 Atlantic–Pacific divergences and phylogeographic patterns in the Caribbean

Evidence for two Atlantic–Pacific divergences is detected within the phylogeny of *E. mayana*, but lack of reliable calibration points (fossils or vicariant events) precludes formal inference of meaningful divergence times. Based on branching patterns and inferred genetic distances, the first Atlantic–Pacific break is indicated by the deep basal divergence (15.5%–19.1% K2P for 12S rDNA) between the *E. mayana* sequence from Pacific Panama reported by Sponer and Lessios (2009) and the clade grouping all of our samples. The second more recent Atlantic–Pacific divergence is represented by the split between the lineages from Venezuela and the Gulf of California (6.6%–9.1% K2P for 12S rDNA; 17.5%–20.6% K2P for COI). Three Atlantic–Pacific divergences were detected in its congener *E. braziliensis*, likely occurring at different times, some of which appear to predate the closure of the Panama Isthmus (Hurtado et al., 2016). Two Atlantic–Pacific divergences are also observed in the phylogeographic patterns of *Ligia* (Santamaria et al., 2014). A complex Miocene–Pliocene geological history associated with the emergence of the Isthmus of Panama appears to have provided multiple opportunities for Atlantic–Pacific vicariance of marine organisms (Bacon et al., 2015).

The sister relationship between *E. mayana* from the Gulf of California clade and one of the Caribbean lineages (Venezuela), to the exclusion of other Pacific (Panama Isthmus) and Caribbean lineages, is somewhat analogous to the pattern observed in the supralittoral isopod *Tylos* (Hurtado et al., 2014). In contrast, in *E. braziliensis*, the Gulf of California clade forms a monophyletic group with a lineage inhabiting the Pacific coast of Costa Rica, which in turn groups with Pacific lineages from Panama and Chile (Hurtado et al., 2016). Gulf of California–Caribbean sister relationships, such as those detected in *E. mayana* and *Tylos*, are not surprising. Most fauna‐rich fossil sediments found in the northern Gulf of California show affinities with Caribbean fauna (Escalona‐Alcázar, Delgado‐Argote, López‐Martínez, & Rendón‐Márquez, 2001), and marine fossils with Caribbean affinities in the Gulf of California date back to Miocene times (Smith, 1991).

Highly divergent lineages of *E. mayana* occur in the Caribbean, implying a long and complex evolutionary history within this basin, likely involving instances of overwater dispersal. Despite sparse sampling and unresolved basal relationships, several preliminary inferences can be made. Evidence of some geographical structuring among three major clades is observed, with one subclade within Clade I found in the western Caribbean, Clade II found in the north‐central part, and Clade III occupying the eastern part. In addition, the island of Puerto Rico harbors members of two divergent clades; a pattern also observed in *Ligia* (Santamaria et al., 2014). The occurrence of two highly divergent lineages in a single large Caribbean island was also observed in *Tylos* from Cuba (Hurtado et al., 2014). In both, *E. mayana* and *Ligia* (Santamaria et al., 2014), despite the geographic proximity of Trinidad to the Venezuelan coast, the lineages sampled from these two localities are highly divergent. The Venezuelan lineage of both *E. mayana* and *Ligia* is also highly divergent from the remaining Caribbean lineages, suggesting an independent history of this region from the rest of the Caribbean for these isopods. Comprehensive sampling of the Caribbean basin and the Pacific coast south of the Gulf of California to Colombia (the southern limit of *E. mayana*) is needed to better assess the phylogeographic patterns described above and whether *E. mayana* harbors further hidden genetic diversity.

### 4.2 Phylogeographic patterns in the Gulf of California

The large divergences among some lineages within the Gulf of California clade, as well as between this clade and the Caribbean lineages, suggest a long history of *E. mayana* in this basin. The occurrence of monophyletic Gulf of California clades containing highly divergent lineages has also been reported in *Ligia* (Hurtado et al., 2010) and *Tylos* (Hurtado et al., 2013). The overall phylogeographic patterns of *E. mayana* in the Gulf of California, however, differ from those observed in the supralittoral isopods *Ligia* and *Tylos* in this region. Nonetheless, several clades of *E. mayana* have similar distributions to clades of these two other isopod taxa.

Each of the three isopod taxa (i.e., *Ligia*,* Tylos*, and *E. mayana*) contains a distinct clade that includes populations from the mainland southern Gulf to the south, in the central Pacific coast of Mexico. In *E. mayana*, Clade A was found on the mainland from Mazatlan, Sinaloa (southern part of the Gulf of California), to Playa CRZ, Michoacan (central Pacific Mexico). In *Tylos*, one highly divergent clade has a similar distribution, spanning the region between Mazatlan and Zihuatanejo (Guerrero), which includes Playa CRZ [i.e., “Clade B” in Hurtado et al. (2013)]. Similarly, a clade of *Ligia* is distributed between Topolobampo and Ixtapa [just south of Zihuatanejo; i.e., localities S17–S25 in Hurtado et al. (2010)]. Another highly divergent lineage of *Ligia* was found in this region, in the localities of Puerto Vallarta and Careyes [i.e., “Clade F” in Hurtado et al. (2010)].

A further phylogeographic similarity among *E. mayana*,* Ligia* (Hurtado et al., 2010), and *Tylos* (Hurtado et al., 2013) is the presence of multiple divergent lineages restricted to the Cape region of the Baja peninsula. Sister clades B and C of *E. mayana* are mainly comprised of lineages found in the Cape region of the Baja peninsula, with the following two exceptions: (1) the Clade B sample from Armenta (BAR; Bahia Concepción), also in the southern Peninsula but further north; and (2) the Clade B sample from Topolobampo (TOP) in the Gulf mainland. Divergence between the lineage from Armenta and the other lineages in Clade B (8.4%–9.2% COI K2P) suggests a relatively old split. A similar divergence (7.3% COI K2P) between these two regions is observed in Clade D, comprised of a lineage from La Paz (LPZ; Cape region) and a lineage from Loreto (LOR), just south of Bahia Concepcion. Sequences of individuals from Topolobampo are highly similar or identical to several individuals from Bahia de La Paz that grouped within Clade B (from Espiritu Santo Island and La Paz), suggesting a recent peninsula to mainland dispersal event.

Phylogeographic patterns indicate the Baja Cape region has been an important center of diversity and endemism for the three isopod taxa, with six highly genetically differentiated and endemic lineages within *E. mayana* (i.e., Clade D LPZ, Clade B LPZ‐CAJ, Clade D ESP‐LPZ, Clade C CAJ, Clade C LPZ, and Clade C ARC‐CBP‐BRR; Figure 3), five within *Ligia* (Hurtado et al., 2010), and three within *Tylos* (Hurtado et al., 2013). Divergent lineages confined to the Cape region have also been observed in other terrestrial organisms (Riddle, Hafner, Alexander, & Jaeger, 2000). *Excirolana mayana* is to our knowledge the first swimming organism for which divergences at the Baja Cape region are reported. Past isolation and a long independent history of the Baja Cape region may have promoted diversification and endemism in this region. Temporal seaways separating the Cape region from the rest of the peninsula have been proposed (Grismer, 1994; Murphy, 1983). In addition, it is hypothesized that the Cape region was the last part of the Baja peninsula to separate from mainland, 4–6 Ma (Carreño & Helenes, 2002; Helenes & Carreno, 1999; Helenes & Téllez‐Duarte, 2002; Larson, Menard, & Smith, 1968; Ledesma‐Vásquez & Carreño, 2010).

Similar to *Ligia* and *Tylos*,* E. mayana* shows a highly divergent clade (Clade EFG) that is restricted to the northern Gulf, where it is widely distributed. In *Ligia*, one of the two most basal lineages found in the Gulf was comprised solely of northern Gulf localities, whereas the other occupies the southern part of this basin (Hurtado et al., 2010). *Tylos* has three divergent lineages exclusive to the northern Gulf [i.e., two in Clade G and one in Clade E in Hurtado et al. (2013)]. Distribution of the subgroupings within *E. mayana* Clade EFG is highly concordant with geography: Clade E in the mainland Guaymas–Kino region; Clade F in the northernmost Gulf; and Clade G in the Bahia de los Angeles area and the central peninsula around Santa Rosalia. Clades E, F, and G have similarities and differences with the lineages of *Ligia* and *Tylos* distributed in these areas (Hurtado et al., 2010, 2013). Populations of *E. mayana* and *Ligia* occupying the Upper Gulf show very shallow divergences, suggesting recent colonization that may have been facilitated by low sea levels (Hurtado et al., 2010). In contrast, populations of *Tylos* in this area can be highly divergent and belong to different clades, potentially reflecting a recent colonization by lineages that diverged elsewhere. The Bahia of Los Angeles area (including the nearby islands) appears to be a biodiversity hot spot for the three isopod genera, because it harbors multiple and highly divergent lineages of each. Isolation and differentiation of populations within islands probably contributed to this diversity. Finally, samples of *E. mayana* from Guaymas are highly similar to the nearby insular localities of Isla Tiburon (i.e., El Tordillo) and Isla Datil, whereas the *Ligia* samples from Guaymas are similar to those of the nearby mainland localities of San Carlos (a bay adjacent to Guaymas) and Kino, but highly divergent from those in Isla Tiburon and Isla Datil, which are in a different clade. In *Tylos*, samples from San Carlos and Isla Tiburon are highly divergent from each other.

Despite similarities in the distribution of several regional lineages, the general phylogeographic patterns of *E. mayana*,* Ligia*, and *Tylos* exhibit marked differences. One contrasting phylogeographic pattern among the three intertidal isopods concerns the distribution of the most divergent lineages in the Gulf. In the case of *Ligia*, one of the two most basal lineages is distributed in the northern Gulf, whereas the other is distributed in the southern Gulf (Hurtado et al., 2010). Such a pattern is not observed in *E. mayana* or *Tylos*. Deeply divergent lineages restricted to either the northern or southern Gulf, however, are observed in *E. mayana* and *Tylos* (Hurtado et al., 2013).

Another major discrepancy observed between *Ligia* and *Tylos* is the phylogeographic affinity of the Cape region endemic lineages. In the case of *Ligia*, the closest relatives of the Cape region lineages are in a clade distributed on the mainland between the southern Gulf and central Pacific Mexico, and their divergence is congruent with geological hypotheses that suggest the Baja Cape region was the last portion of the peninsula to separate from the mainland (Hurtado et al., 2010). In the case of *Tylos*, however, the Cape region lineages are more closely related to clades found further north in the Gulf (Hurtado et al., 2013). In *Tylos*, the clade distributed on the mainland between the southern Gulf and central Pacific Mexico is sister to a clade containing all remaining lineages from the Gulf. In the case of *E. mayana*, the clade distributed between the southern Gulf and central Pacific Mexico (Clade A) forms a basal polytomy with clade BC and clade DEFG. A sister relationship between Clade BC and Clade DEFG would resemble the pattern of *Tylos*, whereas a sister relationship of Clade A and Clade BC would resemble the pattern of *Ligia*. Nevertheless, if the alternative relationship (i.e., A  +  DEFG) was true, the phylogeographic pattern of *E. mayana* would differ from both *Ligia* and *Tylos*.

The incongruent phylogeographic patterns of *E. mayana*,* Ligia*, and *Tylos* in the Gulf of California suggest different vicariance or dispersal histories. Due to its more aquatic nature, *E. mayana* is expected to have a higher dispersal potential than *Tylos* or *Ligia*. Notwithstanding their dispersal limitations, colonization of remote volcanic islands (e.g., Hawaiian archipelago, Galapagos, Azores) by members of the genera *Ligia* and/or *Tylos* (Hurtado et al., 2014; Santamaria et al., 2013) must have required long‐distance dispersal events. Unfortunately, the geological history of the Gulf of California is far from resolved (Helenes et al., 2009; Ledesma‐Vásquez, 2002). Therefore, it is not possible to discern which phylogeographic patterns resulted from vicariance and which from dispersal, except for those instances in which identical or very similar haplotypes are shared by specimens from geographically distant locations, implying recent long‐distance overwater dispersal. In *E. mayana*, we detected evidence for a recent long‐distance (~190 km) dispersal event across the Gulf of California, from Bahia de La Paz, on the Peninsula, to Topolobampo, on the mainland. Hurricanes, which often affect this region, and vessel traffic (e.g., through ballast water) may have facilitated dispersal. La Paz and Topolobampo are major ports with daily exchange large vessels. In its congener *E. braziliensis*, recent exchange of individuals between localities >1,400 Km apart (i.e., Yucatan Peninsula and Caribbean Panama) has been reported (Hurtado et al., 2016). Thus, albeit apparently rare, long‐distance dispersal appears to have occurred in these cirolanid isopods. Long‐distance human‐mediated dispersal, possibly via water ballast, appears to have occurred in other cirolanids (Bowman, Bruce, & Standing, 1981), although this has not been confirmed with molecular data.

### 4.3 Taxonomy

*Excirolana mayana* was originally described from the “Port of Silam” (presumably Dzilam de Bravo) in the Yucatan peninsula (Ives, 1891). According to our results, the individual examined from Dizlam de Bravo (DZI; i.e., topotype) is most closely related to lineages found in the Caribbean coast of Panama and Costa Rica. Our discovery of high levels of apparently cryptic diversity further complicates a historically challenging taxonomy plagued by missing type material (i.e., the description fails to indicate where it was deposited), lack of adequate illustrations, conflicting description of characters, and ambiguity in reported locality names (Brusca et al., 1995). For example, Richardson (1905) reported *E. mayana* in “San Francisco Bay, Lower California,” which is presumably “Bahia San Francisquito, Baja California” (Brusca et al., 1995), a locality within the Gulf that is close to Isla Salsipuedes (SSP in Figure 2). This has led to misidentifications between *E. mayana* and *E. braziliensis*, which have largely overlapping distributions in the East Pacific and Caribbean coasts (Brusca et al., 1995).

The most recent revision by Brusca et al. (1995) indicates that *E. mayana* is widely distributed throughout the Gulf of California and that it is one of the most abundant animals in the bays of this basin, occurring in vast numbers at sandy beaches. Our surveys of *Excirolana* in the Gulf confirm that *E. mayana* is indeed widespread and common. In the Pacific, Brusca et al. (1995) also report *E. mayana* from one locality in the outer Baja Peninsula coast (i.e., Sebastian Vizcaino Bay), as well as in Costa Rica, Panama, and Colombia. In the Caribbean, the identification of *E. mayana* has also been confusing. Menzies and Glynn (1968) reported this isopod in Puerto Rico and indicate this species is probably a pantropical cosmopolitan. However, Bruce (1981) and Kensley (1984) indicate the samples from Puerto Rico were erroneously identified. Dexter (1972) listed *Cirolana mayana* as the most abundant organism on the two Panamanian beaches she examined (one Pacific and one Atlantic), but Glynn, Dexter, and Bowman (1975) indicated it was a misidentification of *E. braziliensis*. Bruce (1986) listed *E. mayana* as a possible Indo‐West Pacific species.

Potential for misidentification may also happen with *Excirolana chamensis* Brusca & Weinberg, 1987; the third *Excirolana* species formally recognized in the tropical/subtropical East Pacific. Brusca and Weinberg (1987) described this species from one locality on the Pacific coast of Panama. They suggest this species has a highly restricted distribution (only two Pacific localities in Panama), indicating they did not find it among the thousands of *Excirolana* specimens examined from more than 100 locations in the eastern Pacific (from the Gulf of California, Mexico, to Chile), plus numerous samples from the Caribbean and Brazil. Recently, however, *E. chamensis* was reported from Bahia La Paz on the basis of morphology (Torres & Lowry, 2011). Despite our extensive surveys within this bay, including several localities and visits during different years, we only found individuals with *E. mayana* morphology, not *E. chamensis*. Given the problems associated with the identification of the East Pacific *Excirolana* species, the record of *E. chamensis* in Bahia La Paz awaits corroboration. Examination of DNA sequences from *E. chamensis* specimens, which are not available, is necessary to establish whether it constitutes an independent lineage from the *E. mayana* and *E. braziliensis* clades, and to assess its geographic range.

As *E. mayana* appears to constitute a complex of cryptic species, its taxonomy needs revision. We conducted a preliminary examination to identify morphological differences among members of the Gulf of California clade and found subtle differences in the shape and setae of the telson of several lineages. In Clade A, the pleonites are subequal in width and length, and the tip has a semicircular shape with short setae (Fig. S2E). In Clade B, the width of the pleonites is 1.5–2 times their length, and the tip has a rounded triangular shape with short setae (Fig. S2F). In Clade C, the pleonites are subequal in width and length, the tip is blunt and rounded with very short setae that are barely visible, and the posterior border is slightly serrated (Fig. S2G). In Clade D, the pleonites are subequal in width and length, the tip is rounded and triangular with very short setae, and the posterior border is smooth (Fig. S2H). The shape and setae of the telson were similar among clades E, F, and G (Fig. S2I and J). The width of the pleonites is 1.5 times their length, and the tip has an oval to triangular shape with short setae. The above preliminary observations on potential differences among lineages need to be examined in further detail. Morphometrics has allowed some discrimination among otherwise morphologically cryptic lineages of *E. braziliensis* (Lessios & Weinberg, 1994), but body shape landmark‐based morphometrics failed to discriminate among highly divergent cryptic lineages of *Ligia* (Santamaria et al., 2016).

### 4.4 Conservation considerations

*Excirolana mayana* is an abundant member of sandy beach communities in the Gulf of California and Caribbean, where it plays an important ecosystem function as a scavenger. This isopod represents another member of sandy beach communities from these regions for which phylogeographic studies have revealed the presence of numerous divergent lineages with very restricted distributions, which constitute multiple evolutionary significant units, some of which may merit species‐level recognition. Therefore, their extirpation from a locality may result in the extinction of unique biodiversity. Direct‐developing invertebrates (expected to have a limited dispersal potential) constitute a large percentage (>50% in some regions) of the intertidal species found on sandy beaches (Grantham, Eckert, & Shanks, 2003); thus, many more members of sandy beach communities are expected to show high levels of highly restricted unique diversity. Unfortunately, sandy beach communities are largely overlooked for conservation efforts, yet they are very susceptible to human and natural factors that cause disturbances in this habitat (Defeo et al., 2009). Given the highly restricted distribution of differentiated lineages, local efforts to preserve sandy beaches are needed. It is also important to confer these differentiated lineages a conservation status, because the current taxonomy erroneously implies that these are widely distributed species and, thus, not of conservation concern. Protection of the unique diversity identified in the isopods *E. mayana*,* E. braziliensis*,* Tylos*, and *Ligia*, for which high levels of endemism are known in the Gulf of California and Caribbean, may benefit other poorly known intertidal invertebrates associated with sandy shores.

## Conflict of Interest

None declared.
