# Supplementary material for: Phylogeographic patterns of a lower intertidal isopod in the Gulf of California and the Caribbean and comparison with other intertidal isopods
Source: Ecol Evol. 2016 Dec 20;7(1):346–57. doi: 10.1002/ece3.2599 (PMC5214745; doi:10.1002/ece3.2599)
Supplement: Supplementary file 2 [file ECE3-7-346-s002.docx]

Fig. S2. Morphological traits of each clade of *E. mayana*. A. Clade II (Caribbean). B. Clade IV (Caribbean). C. Clade III (Caribbean). D. Clade I (Caribbean). E. Clade A (Gulf of California). F. Clade B (Gulf of California). G. Clade C (Gulf of California). H. Clade D (Gulf of California). I. Clade E (Gulf of California). J. Clades F and G (Gulf of California).

**A. Clade II (Caribbean)**

**B. Clade IV (Caribbean)**

**C. Clade III (Caribbean)**

**D. Clade I (Caribbean)**

**E. Clade A (Gulf of California)**

**F. Clade B (Gulf of California)**

**G. Clade C (Gulf of California)**

**H. Clade D (Gulf of California)**

**I. Clade E (Gulf of California)**

**J. Clades F and G (Gulf of California)**
