# Supplementary material for: Phylogeographic patterns of a lower intertidal isopod in the Gulf of California and the Caribbean and comparison with other intertidal isopods
Source: Ecol Evol. 2016 Dec 20;7(1):346–57. doi: 10.1002/ece3.2599 (PMC5214745; doi:10.1002/ece3.2599)
Supplement: Supplementary file 3 [file ECE3-7-346-s003.docx]

Supporting Table S1. Localities Sampled and GenBank Accession Nos. for *Excirolana mayana*. Locality ID corresponds to abbreviated name used in figures.

| **Locality ID** | **Locality Name** | **Country** | **Latitude** | **Longitude** | **Cytb** | **COI** | **12S** | **16S** |
| --- | --- | --- | --- | --- | --- | --- | --- | --- |
| PCH | Pos Chiquito | Aruba |  |  | KT122615 | KT122578 | KT122504 | KT122464 |
| NAS | Western Esplanade Beach, Nassau | Bahamas | 25.08° N | 77.35° W | KT122701 | KT122577 | KT122519 | KT122461 |
|  |  |  |  |  | KT122706 |  |  |  |
|  |  |  |  |  | KT122707 |  |  |  |
|  |  |  |  |  | KT122708 |  |  |  |
|  |  |  |  |  | KT122709 |  |  |  |
| CAH | Cahuita | Costa Rica |  |  | KT122591 | KT122560 | KT122501 | KT122429 |
|  |  |  |  |  | KT122600 |  |  |  |
|  |  |  |  |  | KT122601 |  |  |  |
|  |  |  |  |  | KT122602 |  |  |  |
| ROY | via Port Royal, Jamaica | Jamaica | 17.94° N | 76.82° W | KT122702 | KT122583 | KT122520 | KT122482 |
|  |  |  |  |  | KT122703 |  |  |  |
|  |  |  |  |  | KT122710 |  |  |  |
|  |  |  |  |  | KT122711 |  |  |  |
|  |  |  |  |  | KT122712 |  |  |  |
| ANG | Bahia de los Angeles | Mexico | 28.94° N | 113.55° W | KT122649 | KT122556 | KT122530 | KT122417 |
|  |  |  |  |  | KT122650 |  |  |  |
|  |  |  |  |  | KT122651 |  |  |  |
|  |  |  |  |  | KT122652 |  |  |  |
|  |  |  |  |  | KT122653 |  |  |  |
| ARC | Puerto del Arco | Mexico | 23.9° N | 109.8° W | KT122692 | KT122557 | KT122539 | KT122418 |
| BAR | Bahia Armenta | Mexico |  |  | KT122677 | KT122558 | KT122517 | KT122427 |
| BRR | Los Barriles | Mexico |  |  | KT122671 | KT122559 | KT122531 | KT122428 |
|  |  |  |  |  | KT122672 |  |  |  |
|  |  |  |  |  | KT122673 |  |  |  |
|  |  |  |  |  | KT122741 |  |  |  |
|  |  |  |  |  | KT122742 |  |  |  |
|  |  |  |  |  | KT122743 |  |  |  |
| CAJ | Cajete | Mexico | 24.26° N | 110.61° W | KT122667 | KT122561 | KT122522 | KT122430 |
|  |  |  |  |  | KT122732 | KT122562 | KT122533 | KT122431 |
|  |  |  |  |  | KT122733 |  |  |  |
|  |  |  |  |  | KT122734 |  |  |  |
|  |  |  |  |  | KT122735 |  |  |  |
|  |  |  |  |  | KT122736 |  |  |  |
|  |  |  |  |  | KT122737 |  |  |  |
|  |  |  |  |  | KT122738 |  |  |  |
| CBP | Cabo Pulmo | Mexico |  |  | KT122693 | KT122563 |  | KT122434 |
| COR | Isla Coral | Mexico |  |  | KT122715 | KT122564 |  | KT122437 |
|  |  |  |  |  | KT122760 |  |  |  |
|  |  |  |  |  | KT122761 |  |  |  |
|  |  |  |  |  | KT122762 |  |  |  |
|  |  |  |  |  | KT122763 |  |  |  |
| CRZ | Playa Carrizalillo | Mexico |  |  | KT122675 | KT122565 |  | KT122438 |
|  |  |  |  |  | KT122676 |  |  |  |
|  |  |  |  |  | KT122744 |  |  |  |
|  |  |  |  |  | KT122745 |  |  |  |
|  |  |  |  |  | KT122746 |  |  |  |
| ESP | Espiritu Santo | Mexico |  |  | KT122688 | KT122566 |  | KT122443 |
|  |  |  |  |  | KT122689 |  |  |  |
|  |  |  |  |  | KT122690 |  |  |  |
|  |  |  |  |  | KT122691 |  |  |  |
|  |  |  |  |  | KT122754 |  |  |  |
| GYM | Guaymas | Mexico |  |  | KT122645 | KT122568 | KT122529 | KT122448 |
|  |  |  |  |  | KT122646 |  |  |  |
|  |  |  |  |  | KT122647 |  |  |  |
|  |  |  |  |  | KT122648 |  |  |  |
|  |  |  |  |  | KT122728 |  |  |  |
| ISD | Isla Datil | Mexico |  |  | KT122635 | KT122569 | KT122525 | KT122449 |
|  |  |  |  |  | KT122636 |  |  |  |
|  |  |  |  |  | KT122637 |  |  |  |
|  |  |  |  |  | KT122638 |  |  |  |
|  |  |  |  |  | KT122725 |  |  |  |
| LCO | Las Conchas | Mexico |  |  | KT122683 | KT122570 | KT122538 | KT122450 |
|  |  |  |  |  | KT122684 |  |  |  |
|  |  |  |  |  | KT122685 |  |  |  |
|  |  |  |  |  | KT122686 |  |  |  |
|  |  |  |  |  | KT122687 |  |  |  |
| LOR | Loreto | Mexico |  |  | KT122663 | KT122571 | KT122535 | KT122451 |
|  |  |  |  |  | KT122664 |  |  |  |
|  |  |  |  |  | KT122665 |  |  |  |
|  |  |  |  |  | KT122666 |  |  |  |
|  |  |  |  |  | KT122731 |  |  |  |
| LPZ | La Paz | Mexico |  |  | KT122668 | KT122572 | KT122532 | KT122452 |
|  |  |  |  |  | KT122669 | KT122573 | KT122537 | KT122453 |
|  |  |  |  |  | KT122670 | KT122574 |  | KT122454 |
|  |  |  |  |  | KT122739 | KT122575 |  | KT122455 |
|  |  |  |  |  | KT122740 |  |  | KT122456 |
|  |  |  |  |  | KT122749 |  |  |  |
|  |  |  |  |  | KT122750 |  |  |  |
|  |  |  |  |  | KT122751 |  |  |  |
|  |  |  |  |  | KT122752 |  |  |  |
|  |  |  |  |  | KT122753 |  |  |  |
| MAZ | Mazatlan | Mexico |  |  | KT122634 | KT122576 | KT122513 | KT122460 |
|  |  |  |  |  | KT122674 |  |  |  |
|  |  |  |  |  | KT122723 |  |  |  |
|  |  |  |  |  | KT122724 |  |  |  |
| PUE | Puertecitos | Mexico |  |  | KT122654 | KT122582 | KT122514 | KT122470 |
| SBR | San Bruno | Mexico | 27.16° N | 112.16° W | KT122658 | KT122584 | KT122516 | KT122483 |
|  |  |  |  |  | KT122659 |  |  |  |
|  |  |  |  |  | KT122660 |  |  |  |
|  |  |  |  |  | KT122661 |  |  |  |
|  |  |  |  |  | KT122662 |  |  |  |
| SFL | San Felipe | Mexico |  |  | KT122644 | KT122490 | KT122585 | KT122490 |
| SRO | Santa Rosalia | Mexico | 27.36° N | 112.28° W | KT122655 | KT122586 | KT122515 | KT122494 |
|  |  |  |  |  | KT122656 |  |  |  |
|  |  |  |  |  | KT122657 |  |  |  |
|  |  |  |  |  | KT122729 |  |  |  |
|  |  |  |  |  | KT122730 |  |  |  |
| SSP | Salsipuedes | Mexico | 28.72° N | 112.95° W | KT122679 | KT122587 | KT122534 | KT122495 |
| TOP | Topolobampo | Mexico |  |  |  | KT122536 |  | KT122680 |
|  |  |  |  |  |  |  |  | KT122681 |
|  |  |  |  |  |  |  |  | KT122682 |
|  |  |  |  |  |  |  |  | KT122747 |
|  |  |  |  |  |  |  |  | KT122748 |
| TOR | El Tordillo | Mexico |  |  | KT122694 | KT122589 | KT122518 | KT122497 |
|  |  |  |  |  | KT122695 |  |  |  |
|  |  |  |  |  | KT122696 |  |  |  |
|  |  |  |  |  | KT122697 |  |  |  |
|  |  |  |  |  | KT122755 |  |  |  |
| VIB | Viborita | Mexico |  |  | KT122642 | KT122590 | KT122527 | KT122498 |
| FTS | Fort Sherman | Panama |  |  | KT122592 |  |  |  |
| POR | Portobelo | Panama | 9.54° N | 79.67° W | KT122756 | KT122579 | KT122511 | KT122467 |
|  |  |  |  |  | KT122757 |  |  |  |
|  |  |  |  |  | KT122758 |  |  |  |
|  |  |  |  |  | KT122759 |  |  |  |
| PRN | Bahia Las Cabezas | Puerto Rico | 18.37° N | 65.64° W | KT122618 | KT122580 | KT122505 | KT122468 |
|  |  |  |  |  | KT122619 |  |  |  |
|  |  |  |  |  | KT122620 |  |  |  |
|  |  |  |  |  | KT122621 |  |  |  |
|  |  |  |  |  | KT122622 |  |  |  |
| PRS | Monkey Island | Puerto Rico | 18.16° N | 65.74° W | KT122598 | KT122581 | KT122500 | KT122469 |
|  |  |  |  |  | KT122599 |  |  |  |
| T&T | Chaguaramas Marina, Trinidad | Trinidad and Tobago | 10.68° N | 61.62° W | KT122704 | KT122588 | KT122521 | KT122496 |
|  |  |  |  |  | KT122705 |  |  |  |
|  |  |  |  |  | KT122713 |  |  |  |
|  |  |  |  |  | KT122714 |  |  |  |
| GUA | Playa El Guaro, La Borracha, Anzoategui | Venezuela | 10.29° N | 64.76° W | KT122608 | KT122567 | KT122503 | KT122447 |
|  |  |  |  |  | KT122609 |  |  |  |
|  |  |  |  |  | KT122610 |  |  |  |
|  |  |  |  |  | KT122611 |  |  |  |
| DZI | Dzilam de Bravo, Yucatan | Mexico | 21.40° N | 88.87° W |  |  | KX530941 | KX530937 |
| PER^1^ | Isla Perico | Panama |  |  |  |  | KP184702 |  |

^1^ (Sponer and Lessios 2009)

Supporting Table S2. Description of characters and the best substitution models identified for the concatenated dataset. Number of characters per gene region that were excluded from and included in the phylogenetic analyses. The number of parsimony informative characters is based on included characters only. Best model selected by jModelTest according to each criterion (AIC, AICc, BIC) and its corresponding weight.

| Gene | Samples | Total characters ^a^ | Excluded characters ^ab^ | Included characters | Parsimony informative | AICc (weight) | AIC  (weight) | BIC (weight) |
| --- | --- | --- | --- | --- | --- | --- | --- | --- |
| 16S rDNA | 57 | 453 | 165 | 288 | 104 | TVMef+I+G (0.87) | TVMef+I+G (0.53) | TVMef+I+G(0.87) |
| 12S rDNA | 57 | 486 | 114 | 372 | 130 | GTR+I+G (0.81) | GTR+I+G (0.97) | SYM+I+G (0.44) |
| Cytb | 57 | 309 | 0 | 309 | 155 | TIM3+I+G (0.65) | TIM3+I+G (0.79) | TIM3+I+G (0.57) |
| COI | 57 | 543 | 0 | 543 | 215 | GTR+I+G (0.76) | GTR+I+G (0.92) | TIM2+I+G (0.58) |
| MT | 57 | 1791 | 279 | 1512 | 604 | GTR+I+G (0.99) | GTR+I+G (0.99) | SYM +I+G (0.55) |
|  |  |  |  |  |  |  |  |  |

^a^ Total number of characters in the alignment, including gaps

**^b^** Criteria for character exclusion are described in a nexus file in the supporting information

MT = combined mitochondrial genes

Supporting Table S3. Models, parameters, and priors used in the Maximum Likelihood and Bayesian phylogenetic analyses of the concatenated dataset.

| Method | Model and Priors^1^ | Partitioning scheme^2^ | iterations generations/bootstrap replicates | Sample frequency | runs/ chains | burnin | ASDSF^3^ | Bayes Factors^4^ /ML scores (-lLn) | ESS^4,5^  > 200 | PSRF^6^ |
| --- | --- | --- | --- | --- | --- | --- | --- | --- | --- | --- |
| RaxML | GTR G | 1 | 1000 | na | na | na | na | -16475.625 | na | na |
| Garli | GTR G | 1 | 1000 | na | na | na | na | -15595.338 | na | na |
| MrBayes | GTR G | 1 | 10,000,000 | 1,000 | 4/4 | 25% | 0.002435 | -16337.023 | yes | 1 |
| MrBayes | GTR G | 5(12S+16s+Cytb1,Cytb2+COI2,Cytb3,COI1,COI3)^7^ | 10,000,000 | 1,000 | 4/4 | 25% | 0.0042792 | -15331.528 | yes | 1 |
| Phycas | polytomy prior | 1 | 500,000 | 100 | na | 20% | na | -16341.630 | na | na |
|  |  |  |  |  |  |  |  |  |  |  |
|  |  |  |  |  |  |  |  |  |  |  |

^1^ All others default; ^2^ different partitions separated by comma; ^3^ Average standard deviation of split frequencies; ^4^ estimated in Tracer v.1.5;

^5^ Effective Sample Size; ^6^ Potential Scale Reduction Factor for all parameters; ^7^ Partition finder 1.0 (SYM+I+G; TrN+I; TrN+G; TrN+G; GTR+G)

Supporting Table S4. PCR primers information and annealing temperature (TA).

| **Gene** | **Primer name** | **Primer sequence** | **TA (°C)** | **References** |
| --- | --- | --- | --- | --- |
| 16S rRNA | Crust-16Sf | 5’-GCGACCTCGATGTTGGATTAA-3’ | 48 | (Podsiadlowski and |
|  | Crust-16Sr | 5’-CCGGTCTGAACTCAYATC-3’ |  | Bartolomaeus 2005) |
| 12S rRNA | Crust-12Sf | 5’-CAGCAKYCGCGGTTAKAC-3’ | 50 | (Podsiadlowski and |
|  | Crust-12Sr | 5’-ACACCTACTWTGTTACGACTTATCTC-3’ |  | Bartolomaeus 2005) |
| Cytb | Cytb151F | 5’-TGTGGRGCNACYGTWATYACTAA-3’ | 48-49 | (Merritt et al. 1998) |
|  | Cytb144F | 5’-TGAGSNCARATGTCNTWYTG-3’ |  |  |
|  | Cytb270R | 5’-AANAGGAARTAYCAYTCNGGYTG-3’ |  |  |
|  | Cytb272R | 5’-GCRAANAGRAARTACCAYTC-3’ |  |  |
| COI | HCO-2198 | 5’-TAAACTTCAGGGTGACCAAAAAATCA-3’ | 50-55 | (Folmer et al. 1994) |
|  | LCO-1490 | 5’-GGTCAACAAATCATAAAGATATTGGGG- 3’ |  |  |
|  | M13FLCO-1490 | 5’-TGTAAAACGACGGCCAGTGGTCAACAAA TCATAAAGATATTGG-3’ |  | This study |
|  | M13RHCO-2198 | 5’-CAGGAAACAGCTATGACTAAACTTCAGGG TGACCAAAAAATCA-3’ |  |  |
|  | HCOA | 5’-GRTSNCCNCCNCCYCTRGGRTC-3’ |  |  |
|  | LCOA | 5’-ATTTTGTKTTTGGNGCNTGAGC-3’ |  |  |
