# Supplementary material for: Phylogeographic patterns of a lower intertidal isopod in the Gulf of California and the Caribbean and comparison with other intertidal isopods
Source: Ecol Evol. 2016 Dec 20;7(1):346–57. doi: 10.1002/ece3.2599 (PMC5214745; doi:10.1002/ece3.2599)
Supplement: Supplementary file 1 [file ECE3-7-346-s001.pdf]

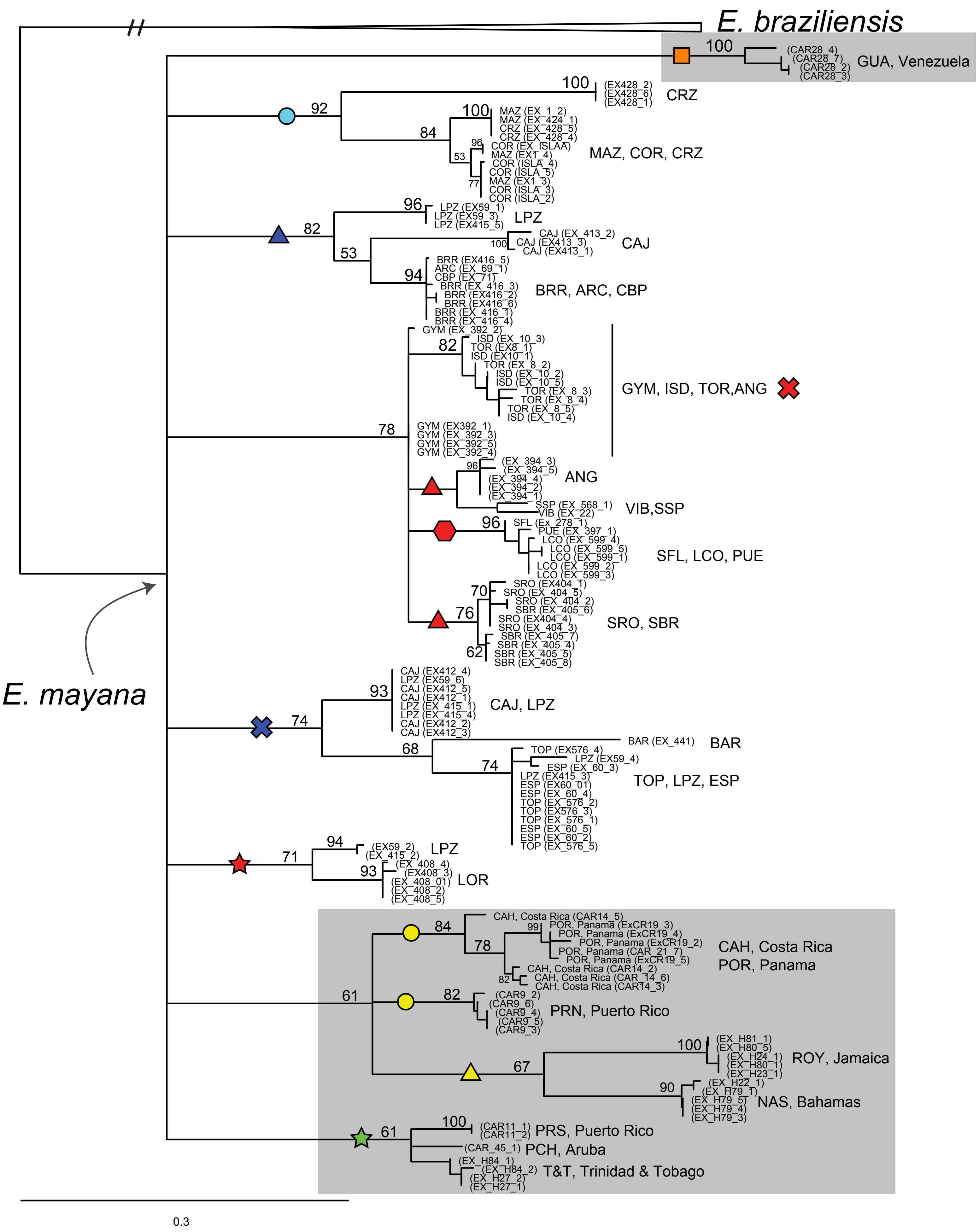

E. braziliensis
100
(CAR28_4)
(CAR28_7)
(CAR28_2)
(CAR28_3)
GUA, Venezuela
100
(EX428_2)
(EX428_6)
(EX428_1)
CRZ
92
100
84
MAZ (EX_1_2)
MAZ (EX_424_1)
CRZ (EX_428_5)
CRZ (EX_428_4)
96
COR (EX_ISLAA)
53
MAZ (EXT_4)
COR (ISLA_4)
COR (ISLA_5)
77
MAZ (EX1_3)
COR (ISLA_3)
COR (ISLA_2)
MAZ, COR, CRZ
96
LPZ (EX59_1)
LPZ (EX59_3)
LPZ (EX415_5)
LPZ
82
CAJ (EX_413_2)
100
CAJ (EX413_3)
CAJ (EX413_1)
CAJ
53
BRR (EX416_5)
ARC (EX_69_1)
94
CBP (EX_71)
BRR (EX_416_3)
BRR (EX416_2)
BRR (EX416_6)
BRR (EX_416_1)
BRR (EX_416_4)
BRR, ARC, CBP
GYM (EX_392_2)
82
ISD (EX_10_3)
TOR (EX8_1)
ISD (EX10_1)
TOR (EX_8_2)
ISD (EX_10_2)
ISD (EX_10_5)
TOR (EX_8_3)
TOR (EX_8_4)
TOR (EX_8_5)
ISD (EX_10_4)
GYM, ISD, TOR,ANG
78
GYM (EX392_1)
GYM (EX_392_3)
GYM (EX_392_5)
GYM (EX_392_4)
96
(EX_394_3)
(EX_394_5)
(EX_394_4)
(EX_394_2)
(EX_394_1)
ANG
SSP (EX_568_1)
VIB (EX_22)
VIB,SSP
96
SFL (Ex_278_1)
PUE (EX_397_1)
LCO (EX_599_4)
LCO (EX_599_5)
LCO (EX_599_1)
LCO (EX_599_2)
LCO (EX_599_3)
SFL, LCO, PUE
70
SRO (EX404_1)
SRO (EX_404_5)
76
SRO (EX_404_2)
SBR (EX_405_6)
SRO (EX404_4)
SRO (EX_404_3)
SBR (EX_405_7)
SBR (EX_405_4)
62
SBR (EX_405_5)
SBR (EX_405_8)
SRO, SBR
E. mayana
CAJ (EX412_4)
LPZ (EX59_6)
93
CAJ (EX412_5)
CAJ (EX412_1)
LPZ (EX_415_1)
LPZ (EX_415_4)
CAJ (EX412_2)
CAJ (EX412_3)
CAJ, LPZ
74
BAR (EX_441)
BAR
68
TOP (EX576_4)
LPZ (EX59_4)
74
ESP (EX_60_3)
LPZ (EX415_3)
ESP (EX60_01)
ESP (EX_60_4)
TOP (EX_576_2)
TOP (EX576_3)
TOP (EX_576_1)
ESP (EX_60_5)
ESP (EX_60_2)
TOP (EX_576_5)
TOP, LPZ, ESP
94
(EX59_2)
(EX_415_2)
LPZ
71
93
(EX_408_4)
(EX408_3)
(EX_408_01)
(EX_408_2)
(EX_408_5)
LOR
84
CAH, Costa Rica (CAR14_5)
99
POR, Panama (ExCR19_3)
POR, Panama (ExCR19_4)
78
POR, Panama (ExCR19_2)
POR, Panama (CAR_21_7)
POR, Panama (ExCR19_5)
CAH, Costa Rica (CAR14_2)
82
CAH, Costa Rica (CAR_14_6)
CAH, Costa Rica (CAR14_3)
CAH, Costa Rica
POR, Panama
61
82
(CAR9_2)
(CAR9_6)
(CAR9_4)
(CAR9_5)
(CAR9_3)
PRN, Puerto Rico
100
(EX_H81_1)
(EX_H80_5)
(EX_H24_1)
(EX_H80_1)
(EX_H23_1)
ROY, Jamaica
67
90
(EX_H22_1)
(EX_H79_1)
(EX_H79_5)
(EX_H79_4)
(EX_H79_3)
NAS, Bahamas
61
100
(CAR11_1)
(CAR11_2)
PRS, Puerto Rico
(CAR_45_1)
PCH, Aruba
(EX_H84_1)
(EX_H84_2)
(EX_H27_2)
(EX_H27_1)
T&T, Trinidad & Tobago
0.3
